# Assistive technologies in employment and entrepreneurship: Graduates with sensory disabilities

**DOI:** 10.4102/ajod.v15i0.1998

**Published:** 2026-05-29

**Authors:** Sibongile Xashimba, Marlene de Beer

**Affiliations:** 1 Department of Social Work and Community Development, Faculty of Humanities, University of Johannesburg, Johannesburg, South Africa

**Keywords:** assistive technologies, unemployment, sensory disabilities, social model of disability, entrepreneurial opportunities, graduates, job seeking, South Africa

## Abstract

**Background:**

Across practice and policy, assistive technologies (ATs) are positioned as a pathway to economic inclusion for graduates with sensory disabilities in South Africa. Still, systemic barriers persist. Developing workable interventions requires in-depth attention to the lived experiences of graduates, which include knowledge on the way devices, systems and attitudes interact in everyday employment seeking and enterprise activities.

**Objectives:**

The study endeavoured to examine how unemployed tertiary-level graduates with sensory disabilities in Gauteng use AT in seeking employment and practising entrepreneurship. Specific enablers and challenges were identified to provide an explanation of variation in economic participation.

**Method:**

Interpretive methods guided the study within a qualitative design. Sixteen participants took part in semi-structured interviews: 10 unemployed graduates (with visual or hearing impairments), three human resources professionals and three helping professionals. Coding and analytic development followed Braun and Clarke’s six-phase thematic approach.

**Results:**

Overall patterns were organised into six themes: (1) discrimination and stigma, (2) accessibility, (3) use and effectiveness of AT, (4) training and support, (5) institutional and government support, and (6) self-initiated strategies and adaptation.

**Conclusion:**

Device access proved insufficient. Most constraints operated at the system level, consistent with the social model of disability, as stigma, accessibility failures, limited training and weak enforcement shaped outcomes. Sustained change had to address stigma, accessibility, training and support and accountable policy implementation through coordinated multi-stakeholder action.

**Contribution:**

By centring graduate voices, this article contributed evidence on adaptability and multi-stakeholder lenses relevant to inclusive policy and practice in resource-constrained settings.

## Introduction

Across practice and policy, assistive technology (AT) is positioned as a pathway to economic inclusion for graduates with sensory disabilities in South Africa. Yet, for many tertiary-level graduates, entry into employment and viable self-employment remains constrained. Assistive technologies may be available, but participation in entrepreneurship and job seeking can still fail when stigma persists, systems are inaccessible and support is discontinuous. Much discussion about AT focuses on provisions effected by the White Paper on the Rights of Persons with Disabilities (Department of Social Development 2016) and the *Employment Equity Act 55 of 1998* (Republic of South Africa 1998). Less attention is paid to the everyday ‘fit’ between tools and the environments in which they must operate: recruitment portals, workplace platforms, routine communication demands and platform-based enterprise activities. A social model lens directs attention to this interaction between impairment and context, including institutional practices and attitudes that can either enable or block AT use in practice.

This article examines how unemployed graduates with sensory disabilities, specifically partial to extreme loss of hearing or vision, in Gauteng use AT while seeking employment and pursuing entrepreneurship. It identifies enablers and challenges that help explain variation in economic participation. The aim is to examine how unemployed graduates with sensory disabilities in Gauteng use AT in seeking employment and practising entrepreneurship. The objectives are to identify key barriers and enabling conditions shaping AT-enabled participation and to derive practical recommendations for strengthening employment access and entrepreneurial participation.

Findings are presented as six themes spanning discrimination and stigma, accessibility, use and effectiveness of AT, training and support, institutional and government support and self-initiated strategies and adaptation.

## Research methods and design

### Study design

Interpretive methods guided the study within a qualitative design. Semi-structured interviews were used to elicit detailed accounts of participants’ experiences and perspectives regarding AT in relation to employment seeking and entrepreneurial activities.

### Setting

The study was conducted in Gauteng, one of the nine provinces in South Africa. Participants lived or worked in urban neighbourhoods spanning Kempton Park, Germiston, Ekurhuleni, Johannesburg, Brackenhurst, Randfontein, Soweto, Centurion, and the executive capital city Pretoria. Gauteng served as the primary location of practice because it combines varied socio-economic issues with differing levels of access to entrepreneurial and employment opportunities.

### Study population and sampling strategy

Overall, a sample of 16 participants was drawn from three key groups: 10 unemployed graduates with sensory disabilities (visually impaired, hard-of-hearing and Deaf participants), three human resources (HR) professionals and three helping professionals (social workers and a psychologist). The intended sample size (*n* = 16) was set to include these three stakeholder groups and to enable triangulation across graduate, recruitment and support-service perspectives. The age profiles of the graduates spanned the late twenties through mid-forties and their qualifications comprised, inter alia, degrees in Visual Arts, Education, Engineering, Computer Science, Marketing, Environmental Studies and Graphic Design. The work experience of HR and the helping professionals ranged from six to more than 15 years.

Inclusion criteria were applied as follows:

- **Graduates:** Participants self-identifying as having a sensory disability (visual impairment, hard-of-hearing and Deaf), holding a post-school qualification, residing in Gauteng and unemployed at the time of participation.
- **HR professionals:** Participants with recruitment-related experience in Gauteng-based organisational contexts.
- **Helping professionals:** Participants providing disability-related support, psychosocial services or disability policy advisers within the study setting.

Groups that were excluded from the study consisted of employed graduates living with or without sensory disabilities, either within or outside of Gauteng, helping professionals with no experience in disability-related support services, and HR professionals not involved in inclusion policy and recruitment.

Sampling was purposive across the three groups, and recruitment included referral pathways where appropriate (Table 1). Bringing these perspectives together strengthened insight and widened interpretation, particularly regarding employment and support experiences of persons with disabilities within local organisational and service-delivery systems.

**TABLE 1:** Participant interview overview.

Number	Interview date	Participant name	Disability	Profession	Age (years)	Race	Gender	Education	Experience (years)	Audio shared date	Transcription-shared date	Participant and/or work location	Individual interview	Activity status
**Unemployed graduates**														
1.	10 January 2025	P1	Visual	-	27	Coloured person	Female	BA Education	-	01 April 2025	01 April 2025	Pretoria, Gauteng	Audio (referral)	Jobseeker
2.	20 January 2025	P2	Visual	-	28	White person	Female	BA Visual Arts	-	01 April 2025	01 April 2025	Centurion, Gauteng	Audio (referral)	Aspiring entrepreneur
3.	21 January 2025	P3	Visual	-	43	Black person	Female	BA Education	-	02 April 2025	02 April 2025	Soweto, Gauteng	Audio	Jobseeker
4.	15 January 2025	P4	Visual	-	36	Not given	Male	Computer Sciences	-	01 April 2025	01 April 2025	Pretoria, Gauteng	Audio (referral)	Jobseeker
5.	20 January 2025	P5	Visual	-	40	White person	Male	BEng Engineering	-	02 April 2025	02 April 2025	Germiston, Gauteng	Audio	Jobseeker
6.	16 January 2025	P6	Hard-of-hearing	-	34	Not given	Male	Sales Marketing	-	N/A	02 April 2025	Johannesburg	Typed feedback	Jobseeker
7.	20 January 2025	P7	Deaf	-	29	Indian person	Male	Graphic Design	-	N/A	02 April 2025	Brackenhurst	Typed feedback	Aspiring entrepreneur
8.	20 January 2025	P8	Deaf	-	45	Not given	Male	Environment Studies	-	N/A	02 April 2025	Ekurhuleni	Typed feedback	Jobseeker
9.	03 February 2025	P9	Hard-of-hearing	-	30	Coloured person	Female	Dip Marketing	-	N/A	02 April 2025	Randfontein	Typed feedback	Jobseeker
10.	06 February 2025	P10	Deaf	-	32	Black person	Male	Civil Engineering	-	N/A	02 April 2025	Kempton Park	Typed feedback	Aspiring entrepreneur
**HR and helping professionals**														
1.	04 February 2025	P11	-	HR	-	Black person	Female	-	6	02 April 2025	02 April 2025	Gauteng	Audio	-
2.	12 February 2025	P12	-	HR	-	Black person	Male	-	+15	03 April 2025	03 April 2025	Johannesburg	Audio	-
3.	22 February 2025	P13	-	Policy adviser	-	Not given	Male	-	+10	03 April 2025	03 April 2025	Gauteng	Audio	-
4.	22 January 2025	P14	-	Social worker	-	Black person	Female	-	+8	N/A	03 April 2025	Johannesburg	Typed feedback	-
5.	01 March 2025	P15	-	Psychologist	-	Black person	Male	-	10	03 April 2025	03 April 2025	Gauteng	Audio	-
6.	14 March 2025	P16	-	Social worker	-	Coloured person	Male	-	+12	03 April 2025	03 April 2025	Gauteng	Audio	-

HR, human resources; N/A, not applicable.

Because of restricted access to Gauteng tertiary disability units and disability community centres, the researcher’s attempt to find participants via those channels was unsuccessful. This resulted in negotiations with the centre manager at a disability community centre in Soweto to act as a gatekeeper in order to distribute the research questions to Deaf and hard-of-hearing participants who frequent the centre. Through accessible professional and social platforms, the researcher made telephonic contact using cold calling techniques to find visually impaired participants, HR professionals and helping professionals. Existing participants willingly referred others to participate in the study.

The researcher facilitated the consent process by obtaining informed consent from each individual before participating in the study. Each participant was informed about the study purpose, the voluntary nature of the participation and their rights. The centre manager played an important role in communicating with Deaf and hard-of-hearing graduates who opted to submit written consent forms. Other participants opted for verbal consent via telephone and WhatsApp voice notes directly to the researcher.

Triangulation of the findings was supported, and credibility was strengthened by the deliberate selection of varied professional experience, educational background, gender and race, thereby enabling analysis of social, technological and structural dynamics shaping AT use across employment and entrepreneurial contexts for graduates with sensory disabilities.

### Data collection

Several modalities supported participation, including WhatsApp voice notes and written responses, as well as telephone interviews. Written formats were additionally provided for Deaf and hard-of-hearing participants. Design choices prioritised inclusion for all participants while still enabling the collection of rich, detailed narratives. Typed feedback and audio recordings, selected to match accessibility needs and preferences, were the methods applied to collect data.

Telephone, WhatsApp and an intermediary were the main avenues through which the interview questions were communicated to participants. These multiple avenues of communication enabled flexibility and prioritised accommodating participants’ different communication requirements.

The researcher was mainly responsible for collecting data, communicating with participants and guiding the centre manager. Interview questions and responses were sent and received mainly through remote communication, data was submitted in writing and audio with all participants communicting in English. The researcher sent consent forms, information letters and interview questions via WhatsApp messaging and conducted telephone interviews with visually impaired participants, as well as HR and helping professionals. The centre manager shared the materials with Deaf and hard-of-hearing participants, who utilised the centre’s computers to type their responses and signed the consent forms, which were then sent back to the researcher in PDF format by the centre manager.

### Data analysis

Braun and Clarke’s (2006) six-phase framework guided the manual thematic analysis approach used to examine the data. Both written and audio-recorded interview responses were combined to develop a single qualitative dataset. Recognition of all three participant groups was maintained because each group contributed differently on the basis of its role and experience with AT. Audio responses were transcribed verbatim, while written submissions were incorporated as provided.

Because of the manageable dataset size, the researcher conducted all phases of the analysis manually, including data familiarisation, inductive coding and theme identification. Following Lincoln and Guba’s (1985) criteria for trustworthiness, the researcher addressed credibility through participant verification, transferability through detailed contextual description, dependability through consistent procedures and confirmability by basing findings on participants’ responses.

### Ethical considerations

Ethical approval was obtained from the University of Johannesburg Faculty of Humanities Research Ethics Committee (ethical approval number REC-01-783-2024). All participants provided consent. To maintain confidentiality, participants are identified using codes (P1–P16), and identifying details were removed from transcripts and reporting. Research data were stored on a password-protected laptop and USB flash drive for backup. To ensure confidentiality, access was restricted to the researcher and the supervisor.

## Results

The three participant groups contributed different perspectives. This resulted in six overarching themes (presented in Table 2) emerging from the analysis, capturing both shared and divergent experiences. In the sub-sections that follow, each theme is developed in detail and supported by illustrative quotations, reflecting the voices of graduates, HR professionals and helping professionals.

**TABLE 2:** Theme development table.

Code	Category	Description	Example quote	Sub-theme	Theme
Employer bias and misconceptions	Employment barriers	Prejudices and assumptions limit the hiring of sensory-disabled graduates.	‘Employers hesitate to hire visually impaired teachers’ (P1)	Societal and institutional bias	Stigma and discrimination
Cost and funding constraints	Financial barriers	High prices and lack of funding limit AT access.	‘The high cost of assistive technologies has always been a problem’ (P16)	Affordability and resource gaps	Accessibility
Incompatibility of digital tools	Tech limitations	Assistive tech is not integrating well with mainstream job platforms or tasks.	‘Currently, the available tools are not well suited for complex programming work’ (P4)	Tool limitations	Use and effectiveness of assistive technologies
Continuous learning	Self-preparedness	Need for graduates to keep up with evolving assistive technology.	‘Continuous training, and online lessons … help them to stand out to potential employers’ (P16)	Graduate self-development	Training and support
Lack of legal mandates	Policy gaps	No enforceable requirements for assistive technology provision.	‘Companies are not required by strong legal mandates to implement AT solutions’ (P13)	Policy and legislative gaps	Institutional and government support
Portfolio development	Self-advocacy	Creating digital portfolios to demonstrate the ability and use of AT.	‘Create portfolios that show their work and how assistive technologies have helped’ (P15)	Positioning for opportunities	Self-initiated strategies and adaptation

AT, assistive technology.

**FIGURE 1:**
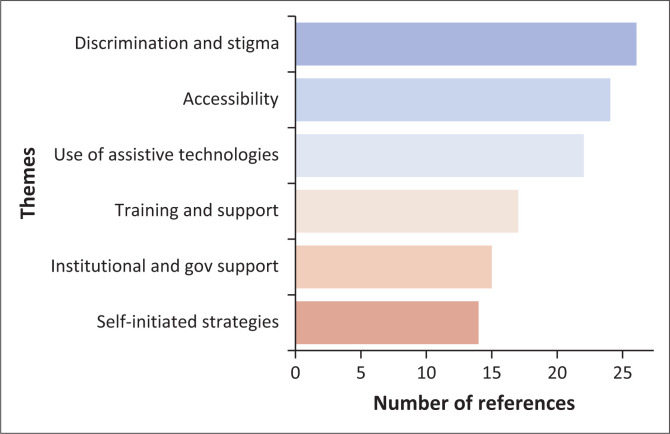
Theme frequency.

Figure 1 provides descriptive context on theme frequency and is used only to orient the detailed qualitative findings that follow.

### Theme 1: Discrimination and stigma

Across the interview accounts, discrimination and stigma surfaced repeatedly, shaping whether AT translated into practical gains in employment and entrepreneurship for graduates with sensory disabilities. South African scholarship continues to report that stigma and employer attitudes can shape recruitment and selection outcomes for persons with disabilities, even where formal commitments to inclusion exist (Morwane 2021).

Functional independence can be strengthened through AT (e.g. screen readers and hearing aids), yet participants described how visible AT use sometimes triggered deficit assumptions that overshadowed utility and narrowed employment and entrepreneurial opportunity despite demonstrated skills. Implications follow for the study’s inquiry into AT as a lever against unemployment: technological advantages may be neutralised when stigma governs who is perceived as ‘capable’ of work or enterprise.

Participants linked visible AT use to why they are passed over during hiring processes, regardless of their qualifications, reinforcing negative stereotypes in employer judgement. In majority-world contexts, structural discrimination may operate through embedded attitudes and informal practices rather than explicit policy exclusion, as argued by Barnes and Sheldon (2010). A graduate explained:

‘However, since the internship ended, navigating the job market has been a challenge.’ (P6)

Another graduate described being excluded from roles for which they were qualified:

‘There is a lot of stigma and negative stereotypes associated with my disability, which makes it difficult for me to find work in my field of marketing.’ (P9)

Participant comments pointed to a shared concern, raised by both HR staff and helping professionals: Implicit bias can sit inside ordinary routines, shortlisting, judgements about ‘fit’ and informal gatekeeping, even when written policies appear neutral. One respondent, working in HR, conceded:

‘Many employers remain unsure about effective assisted technologies … so they hesitate to provide necessary accommodations.’ (P11)

A further account placed significant emphasis on ongoing stigma from employers, even with assistive listening devices:

‘… employers often assume that I will not be able to perform as well as my hearing peers, even though I have proven my capabilities in the past.’ (P9)

The viewpoints of some graduates about the use of AT are shaped by this prejudice across entrepreneurial activity and professional settings, affecting judgements about competence, ‘fit’ and legitimacy even when AT functions as a routine accommodation. Stereotypes in the art sector were described by a creative arts graduate with visual impairment, who explained that inclusion remained limited despite using screen readers and speech-to-text tools to run her online pottery business:

‘Art galleries and creative industries do not provide enough inclusive opportunities for visually impaired artists … there are misconceptions about my abilities as a visually impaired artist, which then limits the opportunities for me.’ (P2)

An online teacher with visual impairment reflected on the social attitudes that continue to constrain her endeavours despite her effective use of AT:

‘The schools rarely provide accessible teaching tools or teaching roles, and I face substantial bias against being a blind educator … despite my educational background and qualifications, securing employment remains a challenge.’ (P3)

Across the two accounts, AT-enabled self-employment – online tutoring, e-learning programmes and e-commerce sales of creative work – did generate alternative income streams. However, both participants stressed that stigma still shaped how their work was valued, funded and supported, even when capability and output were evident. Morwane’s (2021) focus on formal employment therefore captures only part of this story. Within entrepreneurial ecosystems, legitimacy is negotiated through platform visibility, customer trust and informal gatekeeping, so stigma can restrict market entry and scaling even when graduates have the skills and the tools to trade. Device performance was not the bottleneck. Instead, social prejudice undermined ATs’ practical value by recasting adaptations as ‘dependency’ or ‘risk’, rather than as routine accommodation in work and business settings. What mattered most was how disability and AT use were read by others, not what the tools could do in principle.

Postcolonial disability analysis frames stigma as an enduring structural residue of historical exclusion, not merely an individual attitude, which helps explain why discriminatory judgements travelled across employment and enterprise pathways in these accounts (eds. Chataika & Goodley 2024). On the ground, exclusionary decisions reduced the chances that graduates could convert AT use into stable employment or sustained venture formation. Comparable patterns are reported in higher-education contexts, where persistent attitudinal barriers and institutional exclusion can blunt the benefits of AT and constrain participation (Mutanga 2017).

### Theme 2: Accessibility

Access challenges determined the way in which unemployed graduates with sensory disabilities in Gauteng province engaged with entrepreneurial and employment opportunities.

Access mattered. Conceptually, social-model thinking frames disability as produced through inaccessible environments and systems rather than as an individual deficit (Oliver 2013). In this dataset, access extended beyond physical space to include digital platforms, workplace systems and communication processes required for effective use of AT. Emphasis is placed where individuals already have advanced tools (e.g. screen readers and hearing aids), yet success remains constrained when organisational systems and digital environments are inaccessible or poorly designed for AT compatibility.

Across accounts, multiple accessibility barriers disrupted job searching and constrained entrepreneurial activity, often at the point of application, communication or platform-based participation. Employment portals and virtual meeting platforms repeatedly generated exclusion. Evidence from accessibility research shows that inaccessible digital interfaces can create substantial barriers for screen-reader users, restricting independent participation and, in practice, limiting access to opportunities (Lazar, Goldstein & Taylor 2015). One visually impaired graduate explained:

‘… the job portals are not designed with accessibility in mind … they make it difficult for me to apply for jobs independently, and then I would have to then rely on my older brother to assist me to navigate these platforms.’ (P1)

Support-service informants, including policy and psychosocial practitioners, framed the barriers as structural (embedded in organisational systems and norms) rather than as individual shortcomings. As a respondent said:

‘There is a lack of AT solutions tailored to specific industries … which limits opportunities.’ (P14)

Prior South African work by Ndlovu and Walton (2016) on transitions from higher education into employment indicates that structured supports available during study may not translate into workplace systems after graduation, with accessibility gaps re-emerging through recruitment processes, organisational routines and everyday practice. Entrepreneurship was also constrained. One participant running an online pottery business, a graduate with visual impairment, described how platform incompatibility restricted market participation despite effective use of AT:

‘I started an online store to sell my pottery and art pieces, so I use accessible e-commerce platforms and social media to market my work … so job portals are such a nightmare to deal with because there’s a lack of accessibility when it comes to jobs.’ (P2)

Recent scholarship on disability and entrepreneurship argues that AT can strengthen entrepreneurial independence by supporting communication, self-management and day-to-day business functioning. However, the present accounts indicate that inaccessible market infrastructures (e.g. registration flows, listing interfaces and payment processes) can still erode – and in some cases nullify – these practical advantages even when the tools themselves function effectively (Tamzini 2024). Access remains decisive. Rather than implying a simple tool–outcome link, these data point to platform compatibility and ecosystem gatekeeping as constraints that are not consistently foregrounded in entrepreneurship discussions. In educational settings, one participant who teaches online emphasised that the main obstacle lay not in AT itself, but in the wider environment that fails to accommodate it.

‘Assistive technologies improve all the time … it’s the spaces, the societies that we are in that need to keep up and be the ones that improve too, so that we can balance the system.’ (P3)

Universal design advocacy calls for environments and systems to be inherently accessible, thereby reducing reliance on individual adaptation and minimising the need for retrofitted accommodation (Vanderheiden 2000). Affordability and organisational readiness were also emphasised by two hard-of-hearing participants:

‘… small businesses are hesitant to adopt AT due to the perceived costs, so government incentives could help make these technologies more accessible.’ (P9)

‘Hearing aids can be expensive and not everyone can afford them. Employers should be aware of the technologies available to support individuals with hearing impairments …’ (P6)

Limitations in terms of cost and awareness, alongside shortages in trained personnel and the lack of service delivery, continue to be prominent barriers to AT access in many low- and middle-income environments (Tangcharoensathien et al. 2018), and the scale of unmet need and low hearing-aid coverage reported globally reinforce the practical importance of affordability-focused and delivery-oriented responses (Orji et al. 2020).

Across accounts, inaccessible digital processes (recruitment and platform-based trading) repeatedly shifted participation from independent use to reliance on intermediaries or disengagement. These barriers reinforced exclusion in both employment entry and enterprise participation.

### Theme 3: Use and effectiveness of assistive technologies

Graduate accounts described how AT was used to navigate employment and entrepreneurship while also clarifying that inclusion depended on more than tool availability. This theme considers whether AT can mitigate unemployment and limited entrepreneurship among graduates with sensory disabilities in Gauteng, and under what enabling conditions this becomes feasible. Evidence from interviews suggests varied benefits: screen readers, speech-to-text, text-to-speech and hearing aids can enhance independence, yet effectiveness remained conditional on system accessibility, affordability and user training, consistent with South African work showing that structural and institutional barriers can limit the practical benefits of AT even where tools are available (Mutanga 2017). Context mattered.

**FIGURE 2:**
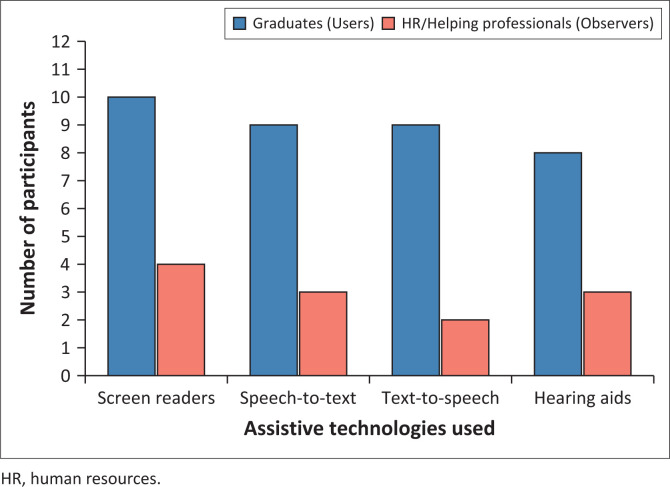
Assistive technologies most frequently discussed across participant groups.

Figure 2 summarises the ATs most frequently discussed across participant groups, providing context for this theme.

**FIGURE 3:**
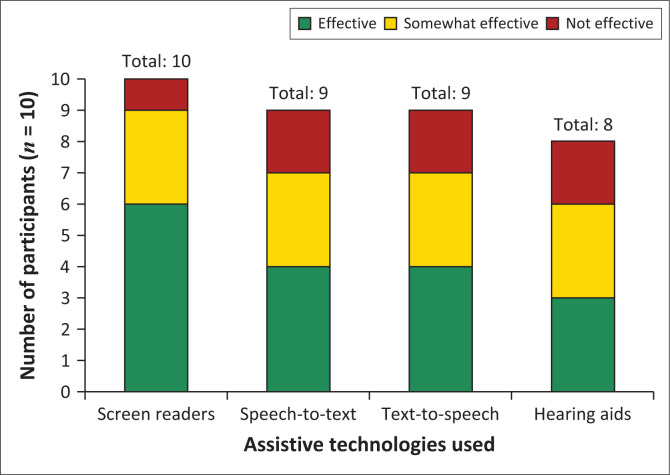
Assistive technology effectiveness.

A summary of participant ratings appears in Figure 3, showing how 10 graduates evaluated the effectiveness of these four ATs on the basis of lived experience across job seeking and enterprise activity. Across the four tools, participants ranked screen readers as most effective, placed speech-to-text and text-to-speech next and described mixed hearing-aid outcomes shaped by cost, device quality and context of use (e.g. workplace communication demands).

Comparable variability has been reported in screen-reader adoption research, which notes transformative potential alongside differences in user experience and system compatibility across settings and stages of use (McCarthy, Pal & Cutrell 2013). Policy perspectives dominated discussion of these tools, with HR staff and helping professionals emphasising gaps in institutional awareness, structured support and practical implementation that would enable AT to function as intended in recruitment, onboarding and daily work.

Across interviews, one tool stood out: screen readers, frequently used and strongly valued by graduates with visual impairment, enabled access to online job advertisements, educational materials and e-learning platforms. In practice, gains in usability became constricted when workplace systems and corresponding software were incompatible with screen-reader input or when employers lacked familiarity with AT-enabled work processes and routines.

Compatibility mattered. This limitation extends the findings of Borodin et al. (2010), who identified screen reader browsing challenges due to inaccessible web design, by situating the problem within South African employment and entrepreneurial contexts. One participant explained:

‘I use screen readers a lot. Audio tools are very helpful in my day-to-day life, and they all navigate digital content and access job postings, and other vital information.’ (P1)

Another graduate who runs an online tutoring business added:

‘I use screen readers text-to-speech technology as well, so it’s almost impossible to just use one. So you find yourself using multiple software and devices … currently I do teaching online so these help me with my remote teaching responsibilities …’ (P3)

Yet even the multiple tools combined were not enough to overcome systemic barriers. A computer science graduate noted:

‘… every day I use screen readers and earlier on much earlier, when I lost my sight in the earlier days, I used to use magnification software and now I use screen readers and text-to-speech technology. This is on a day-to-day basis. OK. While these assistive tools are helpful, I mean. They alone are not sufficient to overcome the barriers and challenges I face when it comes to securing employment in the job market …’ (P4)

What can be learned from these accounts is that screen readers do enhance autonomy, but their potential is curtailed by inaccessible workplace systems and digital platforms; thus their capacity to translate into employment or business outcomes is reduced. In the context of remote or online activities, speech-to-text and text-to-speech functionalities were valued and appreciated for enabling timely communication, supporting the drafting and revision of documents and sustaining productivity when interaction occurred primarily through digitally mediated channels. Recent work on automated recognition using deep learning, including Reddy, Vaishnavi and Kumar’s (2023) research, clarifies the technical foundations that can make these functions more reliable and usable for people with sensory disabilities within digitally mediated work and learning environments. Context determined benefit.

Graduates described using these tools for lesson planning, correspondence and creative projects. A visually impaired participant shared:

‘… these tools to create lessons, you know, lesson plans and communicate with their parents, and this has helped me to build a relationship with my community.’ (P3)

Similarly, a visually impaired artist highlighted how these tools supported her creative and business activities:

‘I use screen readers and speech-to-text software. They’re much easier to use, especially for day-to-day activities, and they just help me express myself more effectively. When it comes to looking for a job they are helpful to a certain extent to a certain extent. I think that I have had challenges with online or just navigating digital spaces …’ (P2)

Helping professionals observed that even when AT is available, many graduates lack adequate technical literacy to use them to their full potential. One participant explained:

‘Many graduates experience digital literacy challenges that make it difficult to navigate AT software. Even if they acquire the technology, they often lack the necessary skills to use it effectively.’ (P14)

Technology-acceptance research identifies training and technological literacy as key conditions for confident adoption and routine integration of tools under workplace constraints (Moodley et al. 2020). Deaf and hard-of-hearing participants viewed hearing aids and captioning as essential, yet affordability, environmental noise and employer attitudes often reduced effectiveness. This aligns with global evidence of substantial unmet hearing-aid need and persistent access barriers, especially in resource-constrained settings (Orji et al. 2020). A participant reflected as follows:

‘To communicate effectively, I use a variety of assistive tools such as hearing aids, sign language interpretation, and written communication. Hearing aids are particularly helpful in one-on-one settings … in larger meetings or virtual interviews, I depend on real-time captioning software.’ (P10)

Participant accounts indicate that AT can enable employment and entrepreneurship (e.g. online tutoring and e-commerce), but gains were often constrained by inaccessible systems, limited employer awareness and inadequate training. These findings present optimistic global evidence on AT-related employment potential (Kruse et al. 2024) and support a social model reading of systemic, not individual, disablement (Oliver 2013). One participant interpreted this tension as ‘technology keeps improving, it’s society that needs to catch up’. Figure 3 summarises graduate ratings of perceived AT effectiveness across employment seeking and enterprise contexts.

### Theme 4: Training and support

Post-university transition exposed uneven preparation for AT use and a weakly structured support pathway (e.g. refresher guidance, troubleshooting and follow-up), which shaped how confidently graduates could apply AT within real work-seeking and enterprise contexts. For graduates, competent tool use underpins employment and entrepreneurial success. In the absence of continued guidance and structured support, even empowering tools may be under-used, inconsistently applied or used in ways that reduce intended benefit. Accordingly, evidence indicates that training and follow-up are necessary conditions for AT to contribute to reduced unemployment and increased entrepreneurship. Access to devices alone seldom translates into inclusion without guidance, preparation and ongoing problem-solving support.

Participant narratives indicate that many received AT training during study, but it focused on academic tasks (accessing content and submitting assignments) rather than workplace routines or business applications (client communication, recruitment platforms, transactions and organisational systems). Technology-acceptance research suggests that technology literacy and training shape sustained use; narrow training leaves graduates underprepared for post-graduation implementation demands (Moodley et al. 2020).

Transfer beyond academia remained uneven. An interviewee put it succinctly:

‘I received good training for school because the assistive training that I received at school was for me to get through school, so it was specifically designed for the university and what I was studying at the university. So it wasn’t necessarily designed for the real world.’ (P2)

Similarly, another noted that although initial training was beneficial, post-graduate support was virtually non-existent:

‘The training that I received in assistive technologies was at school. It was helpful. It provided me with the skills to use screen readers and other basic tools which helped me to complete my assignments and navigate to the school environment, you know, but I must say though, the training could not prepare me for the challenges.’ (P1)

Several participants described how they continued teaching themselves online or through peer networks to remain digitally competent. A visually impaired graduate, for instance, explained how she built on her earlier instruction to sustain an online tutoring venture:

‘… even after school, I did not stop training. I continued with online training programmes to keep me abreast with necessary upgrades because I am aware that technology is ever evolving.’ (P3)

Scholarship emphasising continuous learning and entrepreneurial self-efficacy provides a useful lens for interpreting the self-directed upskilling described here. Nevertheless, participant accounts also expose the inadequacy of formal post-university support structures (Dakung et al. 2023).

Feedback received from the interviewees pointed not only to policy gaps but also to resilience, as HR professionals discussed graduates’ reliance on self-directed learning to sustain the use of AT beyond a higher education environment. The suggestion of one specific participant, an HR consultant, was unambiguous:

‘Universities should team up with employers and create learning spaces that are equipped with assistive technologies that closely mirror the real-world work environments and facilities that students will encounter after graduation.’ (P11)

Pre-employment transition research identifies weak education–workplace collaboration as a persistent barrier, recommending staff preparation and targeted training to strengthen partnerships (Lau et al. 2024). Employer induction seldom included orientation on AT use, and graduates described having to explain their tools, accommodation needs and preferred communication practices while simultaneously trying to demonstrate competence. A participant noted this frustration as follows:

‘Unfortunately, assistive technologies have limited capacity to address the rigid and inaccessible recruitment processes maintained by some employers, which fail to accommodate the needs of individuals with sensory disabilities.’ (P10)

Workplace-based qualitative evidence indicates that limited managerial awareness and insufficiently tailored workplace adjustment processes (including onboarding) can operate as key barriers for employees with hearing impairment (Svinndal, Jensen & Rise 2020).

Limited post-graduation support pushes many graduates towards informal communities, YouTube tutorials, and trial-and-error learning to stay employable and to keep pace with changing digital platforms. Confidence can erode quickly in professional settings when assistance is intermittent or unavailable. Work pace also slows when repeated troubleshooting replaces task completion, reducing productivity and increasing the cognitive load of ‘coping’ at work. Workplace capability to integrate AT remained limited, and HR informants indicated that internal expertise was uncommon. This exposes a systemic gap between tertiary preparation, labour-market expectations and entrepreneurial ecosystems.

When support was absent or delayed, graduates’ self-directed learning became an enabling resource. Over time, iterative adjustment of practices, tools and platform settings helped some build digital skills, trial e-commerce options and run online classes. These adaptive strategies foregrounded entrepreneurship, with purposeful AT use despite systemic constraints (Altschwager 2022). However, self-directed learning should supplement, not replace, structured institutional support. Digital-skills investment must be shared by higher education, government and employers across recruitment, onboarding, platform trading and workplace communication.

### Theme 5: Institutional and government support

Effective AT adoption requires coordinated action among employers, educational institutions and government, with clear implementation roles and follow-through. South African entrepreneurship upskilling research shows that weak business networks, limited awareness of support centres and uneven training and resource access constrain entrepreneurs with disabilities, reinforcing this coordination imperative (Maziriri, Madinga & Lose 2017).

Formal frameworks exist, including the White Paper on the Rights of Persons with Disabilities (Department of Social Development 2016) and the *Employment Equity Act 55 of 1998* (Republic of South Africa 1998), yet uneven implementation and fragmented responsibilities can limit the enabling potential of AT for employment and entrepreneurship when accountability is not shared consistently across duty-bearers and partners. Coordination therefore becomes an operational requirement, not a rhetorical aspiration. Implementation gaps were emphasised by a policy adviser, who described a persistent disconnect between policy intent and day-to-day practice. A practitioner noted:

‘Policy gaps mean that assistive technology adoption is often voluntary rather than mandatory, leading to inconsistent implementation across workplaces … when it comes to making provision for us as the technologies their policy gaps that are not explicit about the adoption of assistive technologies, and it’s causing inconsistency.’ (P13)

Policy implementation critiques within South African public-service scholarship highlight significant deficits in disability-related employment policy, even where legislative frameworks appear strong (Majola & Dhunpath 2016). In addition, a participant (a policy adviser) emphasised the following:

‘South African laws should require that companies conduct AT accessibility audits … they need to be within the South African legal framework in order for the country to be able to promote and create more inclusive hiring.’ (P13)

Across participant accounts, institutional accountability was weakened by a persistent gap between policy intent and practice. Oversight remained inconsistent. In many cases, organisational goodwill determined adherence when oversight was weak.

Several graduates and professionals described absent structured funding mechanisms to support AT provision, maintenance and replacement over time. A graduate commented:

‘I think additionally government incentives could help offset the costs of implementing these techs.’ (P5)

Affordability and life-cycle costs (purchase, fitting, maintenance, repairs and replacement) remain widely reported barriers to hearing ATs in low- and middle-income contexts, even where need is well recognised (McPherson 2014). Maintenance costs were often the tipping point.

Financial constraints translated into fewer opportunities, as graduate interviewees linked costs directly to reduced participation in work-seeking and enterprise activity. In a participant account, unclear government funding channels for young entrepreneurs with disabilities meant that self-funding became the only viable option for sustaining her creative business:

‘I wish the government would be more clear about funding opportunities because I struggled. Hence, I opened an online store instead … I struggled to locate the necessary resources from the government.’ (P2)

A ‘post-education AT gap’ can be observed here, where graduates lose access to institutional supports and encounter opaque funding pathways during transitions into work or entrepreneurship (Mahama, Tutu & Owusu-Bempah 2025). Funding clarity became a practical fault-line. Another graduate reinforced the argument, stating that systemic change must extend beyond technological access to institutional transformation:

‘It’s the spaces, the societies that we are in that need to keep up and be the ones that improve too, so that we can balance the system.’ (P3)

Interview accounts emphasised coordinated government action, including incentives for inclusive employers, funding for local AT innovation and maintenance and mandated annual disability-inclusion reporting to enable comparability over time. Given the evidence of fragmented support for entrepreneurs with disabilities (Maziriri et al. 2017; Sefotho 2017), AT enablement should be approached as socially embedded responses and networked across universities, employers and government. When any node fails, opportunity contracts despite functional devices.

### Theme 6: Self-initiated strategies and adaptation

Where post-graduation institutional and policy support was intermittent, many graduates with sensory disabilities in Gauteng demonstrated strong self-initiative and adaptability in using AT to navigate exclusionary environments. Tamzini (2024) similarly reports agency, resilience and adaptability among disabled entrepreneurs, noting that AT use can strengthen independence and self-confidence even amid accessibility constraints. These practices indicate that AT can open livelihood pathways through self-employment, informal learning and community engagement, particularly when combined with local networks and iterative problem-solving. Recommendations therefore focus on supports that sustain self-initiated strategies rather than leaving them improvised.

Online tutoring and e-learning initiatives became a practical fallback for several graduates with visual impairment after repeated difficulty securing formal work. Assistive tools enabled lesson design, communication with students and management of virtual classrooms, including through screen readers, text-to-speech software and accessible online platforms:

‘I offer online tutoring lessons to students in my community … using these tools to create lessons, lesson plans, and communicate with parents.’ (P3)

Vader et al.’s (2024) study of self-employed individuals with visual impairments offers a close parallel, emphasising resilience and self-advocacy in AT-enabled business settings. The present accounts retain that emphasis while locating it within Gauteng’s resource-constrained realities and uneven enabling conditions. Resource constraints amplified this. Several participants described establishing creative or retail businesses through online channels, producing and selling handmade products or artistic content via e-commerce and social-media visibility mechanisms:

‘I started an online store to sell my pottery and art pieces, using accessible e-commerce sites and social media to market my work.’ (P2)

Matching AT with reliable digital access and purposeful entrepreneurial intent made independent income generation more plausible. This, in turn, enabled graduates to participate economically beyond conventional employment channels through online e-commerce, tutoring and platform-based service delivery. Upholding employability frequently demanded constant, self-directed upskilling in the use of AT, particularly as platforms, accessibility features and employer-facing systems changed over time. Formal support rarely followed. Practical advice captured this expectation:

‘Take courses on inclusive entrepreneurship and accessibility. These courses can provide valuable insights and practical strategies into how to start and run a business that is accessible and inclusive to all individuals.’ (P7)

‘… my advice to graduates with sensory disabilities is that they should learn additional skills to supplement job prospects, learning new programming languages or gaining certifications in their respective field, but it’s important to learn additional skills because you really want to stand out.’ (P4)

Helping professionals agreed that coaching and awareness must extend beyond individual users to colleagues and employers, ensuring shared competence in AT utilisation:

‘I would advise them to get additional coaching in industry-specific AT tools to better position themselves in the competitive job market.’ (P11)

Another participant stated:

‘I would advise them to stay updated on the latest AT advancements that can improve job prospects. Additionally, networking with advocacy organisations that promote disability inclusion can provide valuable resources and support.’ (P14)

Mentoring relationships and collaborative learning environments can sustain inclusive workplace practice by supporting onboarding, day-to-day workplace navigation and confidence-building for graduates with sensory disabilities, particularly through peer mentoring and structured opportunities for shared problem-solving. Creative strategies included written messaging, captioned content and real-time sign-language applications for effective participation in professional and social interactions.

In workplace interactions, the approaches supported personal engagement and also modelled inclusive practice for others, contributing to greater awareness of communication diversity and more routine acceptance of accommodation within teams and informal networks.

These findings inform the integrated recommendations presented in the *Discussion* section.

## Discussion

Across participant accounts, a consistent practical point emerged: access to a device alone was insufficient when tools did not integrate cleanly with recruitment workflows, workplace systems and entrepreneurial tasks (applications, interviews, onboarding systems and platform-based trading).

Stigma and discrimination remained a persistent constraint on AT-enabled participation. In workplace contexts, embedded stereotypes shaping attitudes and behaviour can interact with discriminatory practice to limit how graduates with sensory disabilities access employment and entrepreneurship (Link & Phelan 2001). Participants described hiring bypasses, informal exclusion and scepticism about competence. Screen readers and speech-to-text software were sometimes read as signals of reduced capability, and hearing-aid users reported exclusion from everyday networks. Where AT performance gains were not recognised, assumptions about dependence and ‘extra effort’ influenced recruitment and team integration. Such patterns persisted despite the *Employment Equity Act 55 of 1998* (Elshemy et al. 2024; Sonday 2021).

Accessibility functioned as a gatekeeper for translating skills and AT into routine participation. Across interviews and coding outputs, accessibility barriers constrained both employment entry and entrepreneurial engagement for graduates with sensory disabilities. Participants reported inaccessible recruitment portals and application workflows, workplace systems lacking screen-reader compatibility and uneven platform support. Where adaptation existed, it was inconsistent and often treated as optional. Two linked dimensions emerged: external barriers (e.g. employer systems) and internal constraints related to affordability and awareness. Cost frequently determined whether accessible software could be used, even when technically appropriate, highlighting a policy–practice gap. Participants also emphasised social accessibility (communication, flexibility and employer attitudes). These findings position accessibility as a systemic issue requiring enforcement and coordinated action across government, employers and technology developers.

AT use was experienced as both enabling and fragile. Across graduate narratives, AT supported participation, but perceived effectiveness varied substantially among graduates with sensory disabilities. Job applications, independent study and business activities were feasible when AT use aligned with task demands and platform requirements, reducing reliance on others. Post-graduation, older devices, software updates and weak compatibility with recruitment portals and workplace systems created recurring friction, especially where technical support was absent, delayed or difficult to access. University-ready setups did not consistently transfer into employment contexts because integration and everyday adoption were uneven across teams and organisational routines. The findings therefore indicate a tension between empowerment and dependency, with effectiveness relational and context-dependent. Literature on AT adoption similarly cautions that provision alone is insufficient: sustained benefit requires training, ongoing support and contextual adaptation (Alper & Raharinirina 2006).

Training and support functioned as the bridge between device access and sustained capability. Availability of training and practical support structures strongly shaped AT effectiveness, particularly where tools had to interface with recruitment portals, workplace software and routine communication demands. Participants described AT training as limited, often once-off and largely oriented towards academic tasks rather than workplace routines, client-facing communication or business administration. After graduation, structured support frequently fell away, shifting reliance to self-teaching, peer networks and informal online tutorials to manage updates, compatibility failures and platform changes. Evidence across contexts indicates that provision alone rarely yields sustained benefit: systematic reviews identify inadequate user training, limited support and weak follow-up/maintenance as recurring barriers (Boot et al. 2018; Visagie et al. 2020). Higher education can enable learning, but training and institutional systems are not consistently designed for professional practice, and new barriers often emerge during the transition to work (Ndlovu 2021; Ndlovu & Walton 2016). Enterprise participation was similarly shaped by whether training covered entrepreneurship-facing routines and whether context-relevant support was available.

At institutional and government levels, the findings reinforce that policy intent must be converted into measurable implementation. Institutional and government-level support proved decisive in shaping whether graduates with sensory disabilities could access, use and sustain AT for employment and entrepreneurship, particularly through funding continuity, enforceable inclusion duties and routine monitoring. Legislative and policy frameworks, including the *Employment Equity Act 55 of 1998* (Republic of South Africa 1998) and the White Paper on the Rights of Persons with Disabilities (Department of Social Development 2016), provide a rights-and-duty basis for inclusion and reasonable accommodation, yet participant accounts described uneven implementation and diffuse accountability.

Prior scholarship supports this interpretation: within South African higher education, disability-inclusion policies exist, yet implementation remains uneven, and barriers can carry into labour-market transition (Chiwandire & Vincent 2019; Mutanga 2018). Global disability evidence warns that when governments do not establish structured, transparent AT financing and delivery pathways, affordability and access can remain out of reach for marginalised groups, thereby reproducing inequality (World Health Organization & World Bank 2011). Employment-focused inclusion efforts also require attention to enterprise participation, given that entrepreneurship forms part of the right to work and employment (United Nations 2006:art. 27). Without alignment, a ‘double exclusion’ can emerge where inaccessible workplaces constrain employment entry while under-resourced business-support systems restrict enterprise development (Department of Social Development 2016; *National Small Business Act 102 of 1996*).

From my university-facing standpoint, I initially interpreted these findings through an institutional lens, assuming stronger continuity of post-graduation support than participants later described. Listening closely shifted interpretation towards systemic shortcomings requiring accountability, coordination and follow-through, consistent with reflexive quality practices. Measurable implementation, rather than aspirational language, is therefore non-negotiable, including life-cycle financing (procurement, maintenance, repairs, upgrades, replacement), transparent indicators and reporting and cross-sector collaboration bridging higher education, workplaces and entrepreneurial ecosystems.

Graduates’ self-initiated strategies highlight both agency and systemic shortfall. Participants described navigating constraints by networking informally, building digital skills through online platforms, using low-cost or free tools, and repurposing mainstream technologies (e.g. smartphones with built-in accessibility features) when specialised AT was unavailable or unaffordable (Iytha, Tiwary & Augustine 2024; Soares & Benetti 2025; World Health Organization 2024) Reindal’s (2008) social-relational model frames such agency as contextually situated negotiation of disabling relations, while African disability scholarship similarly links weak institutional or state support to improvised ‘survival’ strategies (Chataika 2012). In this study, adaptation functioned as coping and as resistance, with graduates redesigning routines and re-routing access where systems were not built to include them. However, participant accounts also indicate a risk: emphasising ‘coping’ can shift responsibility from institutions to individuals. Disability scholarship cautions that ‘overcoming’ narratives may obscure structural failures and normalise exclusionary practice (Shakespeare 2013). Resilience, therefore, reflected both capability and inadequate support.

Implications for policy and practice follow directly from this synthesis. Participant accounts point to practical measures designed to strengthen employment access and entrepreneurial participation for graduates with sensory disabilities, with particular emphasis on making AT-enabled pathways workable beyond university settings:

- Sustained AT life-cycle financing (procurement, maintenance, repairs, upgrades, replacement) with transparent eligibility, turnaround times and communication.
- Enforceable accessibility compliance in recruitment portals, onboarding systems and core workplace platforms, supported by routine auditing.
- Structured bridging support post-graduation (accessible onboarding, troubleshooting routes, helpdesk access, peer learning) so that capability is not lost when institutional support falls away.
- Inclusive coaching for employers and co-workers on AT adoption and reasonable accommodation in everyday work.
- Enterprise-facing incubation and micro-grant schemes that reduce start-up and maintenance costs for AT-enabled ventures, alongside formalised mentorship and peer-network programmes.
- Sustained public-awareness initiatives that profile AT-enabled entrepreneurs to challenge stigma, normalise capability and strengthen opportunity recognition.

Coordination was repeatedly emphasised as the enabling mechanism. Hephapreneurship, an entrepreneurship model conceptualised by Sefotho (2017), frames these proposals as community-driven and socially embedded support systems in which relational capability (networks, institutional partnership and local enabling conditions) is treated as central to entrepreneurship for marginalised groups rather than as an optional ‘add-on’ to individual effort.

Limitations should be acknowledged. The study draws on qualitative accounts from a purposive sample in Gauteng and therefore offers depth and analytic transferability rather than statistical generalisation. The findings are shaped by the perspectives of the participants included, and they do not capture the full diversity of sectoral contexts, organisational cultures or AT configurations across provinces. Future research could test bridging-support models in workplace and enterprise settings, evaluate accessibility of recruitment and platform infrastructures in practice and assess the costs and outcomes of life-cycle financing approaches for AT provision and maintenance. Taken together, the findings indicate that ATs matter, but they do not operate in a vacuum.

When stigma, inaccessible systems and discontinuous support persist, AT-enabled capability is converted into extra labour by graduates rather than into durable inclusion. The locus of change, therefore, sits with systems – financing, enforcement, training, platform design and workplace practice – so that graduates’ agency becomes a complement to inclusion, not its substitute.

## Conclusion

Across Gauteng, participant accounts show that AT can enable participation, but device access alone rarely translated into stable employment or sustained enterprise activity. Outcomes depended on whether recruitment systems, workplace platforms and routine communication practices were accessible and whether training, maintenance and support continued after graduation. Where these conditions were absent, graduates relied on workarounds, self-funding and self-directed learning, which sustained some entrepreneurial activity but also reproduced a policy–practice gap in employment inclusion. The findings therefore indicate that meaningful AT-enabled inclusion requires coordinated implementation across higher education, employers and government, including enforceable accessibility, life-cycle financing and practical support pathways that make participation routine rather than exceptional.
